# Higher cardiorespiratory fitness levels are associated with greater hippocampal volume in breast cancer survivors

**DOI:** 10.3389/fnhum.2015.00465

**Published:** 2015-08-26

**Authors:** Laura Chaddock-Heyman, Michael J. Mackenzie, Krystle Zuniga, Gillian E. Cooke, Elizabeth Awick, Sarah Roberts, Kirk I. Erickson, Edward McAuley, Arthur F. Kramer

**Affiliations:** ^1^Department of Psychology, The Beckman Institute for Advanced Science and Technology, University of Illinois at Urbana-ChampaignUrbana, IL, USA; ^2^Department of Behavioral Health and Nutrition, College of Health Sciences, University of DelawareNewark, DE, USA; ^3^School of Family and Consumer Sciences, Texas State UniversitySan Marcos, TX, USA; ^4^Department of Kinesiology and Community Health, University of Illinois at Urbana-ChampaignUrbana, IL, USA; ^5^Department of Psychology, University of PittsburghPittsburgh, PA, USA

**Keywords:** aerobic fitness, brain, cancer treatment, memory, physical activity

## Abstract

As breast cancer treatment is associated with declines in brain and cognitive health, it is important to identify strategies to enhance the cognitive vitality of cancer survivors. In particular, the hippocampus is known to play an important role in brain and memory declines following cancer treatment. The hippocampus is also known for its plasticity and positive association with cardiorespiratory fitness (CRF). The present study explores whether CRF may hold promise for lessening declines in brain and cognitive health of a sample of breast cancer survivors within 3 years of completion of primary cancer treatment. We explored the role of cardiovascular fitness in hippocampal structure in breast cancer survivors and non-cancer female controls, as well as performed a median split to compare differences in hippocampal volume in relatively higher fit and lower fit cancer survivors and non-cancer controls. Indeed, CRF and total hippocampal volume were positively correlated in the cancer survivors. In particular, higher fit breast cancer survivors had comparable hippocampal volumes to non-cancer control participants (Cohen’s *d* = 0.13; *p* > 0.3), whereas lower fit breast cancer survivors showed significantly smaller hippocampal volumes compared to both lower fit and higher fit control participants (Cohen’s *d* = 0.87, *p* < 0.05). These results are the first to identify that CRF may protect the brain health of breast cancer survivors within 3 years of treatment. The present study uniquely contributes to the field of cancer and cognition and emphasizes the importance of investigating how individual differences in CRF play a role in brain changes of breast cancer survivors.

## Introduction

Breast cancer treatment is associated with declines in brain and cognitive health (Pereira Dias et al., 2014). Decreases in performance on tasks of executive function, memory, learning, reasoning, concentration, and attention (Argyriou et al., 2011; Wefel et al., 2011) are often reported post-treatment in breast cancer survivors, coupled with declines in gray matter volume (de Ruiter et al., 2012), poorer white matter health (Ferguson et al., 2007; Abraham et al., 2008; Deprez et al., 2011), and altered brain function (Kesler et al., 2009, 2013b; de Ruiter et al., 2011; Bruno et al., 2012). Given an estimated 232,000 new cases of breast cancer diagnosed in the United States in 2013 (DeSantis et al., 2014), it is important to establish interventions to reduce the risk of these impairments and to enhance the cognitive vitality of breast cancer survivors.

The hippocampus, known for its plasticity, has been shown to play an important role in brain and memory declines following cancer treatment (Kesler et al., 2013a). Indeed, a number of human studies show that breast cancer survivors have smaller hippocampal volumes, which are associated with poorer memory performance, relative to women without cancer (McDonald et al., 2010; Bergouignan et al., 2011; de Ruiter et al., 2011; Kesler et al., 2013a). Animal models suggest that microenvironmental changes associated with cancer treatments may lead to the suppression of cell proliferation, neurogenesis, angiogenesis, and the release of neurotrophic factors in the hippocampus, thereby disrupting plasticity (Dietrich et al., 2006; Winocur et al., 2006; Seigers et al., 2008, 2009; Dietrich, 2010; Hyrien et al., 2010; Seigers and Fardell, 2011). For example, in rodents, administration of chemotherapy agents used for breast cancer have been associated with reduced hippocampal neurogenesis (Seigers et al., 2008; Janelsins et al., 2010), hippocampus-related cognitive deficits (Foley et al., 2008; Walker et al., 2011), and reduced levels of brain-derived neurotrophic factor (BDNF) in the hippocampus (Mustafa et al., 2008), a molecule known to be a mediator of neurogenesis and critical for memory formation (Figurov et al., 1996; Kang and Schuman, 1996; Pang et al., 2004). Radiation treatment has also been shown to impair cognition (Shibayama et al., 2014), decrease the production of new neurons, increase cell apoptosis in the hippocampus (Peissner et al., 1999; Tada et al., 2000; Monje et al., 2002), and disrupt microvascular angiogenesis (Monje et al., 2002; Villeda et al., 2011).

An important next step is to use this understanding of brain and cellular mechanisms to determine methods to offset declines in hippocampal structure and function in cancer survivors following treatment. Cardiorespiratory fitness (CRF) and aerobic exercise are known to positively contribute to neural plasticity of the hippocampus in humans. That is, younger and older healthy humans with higher CRF levels have larger hippocampal volumes (Erickson et al., 2009; Chaddock et al., 2010; Bugg and Head, 2011), and greater hippocampal volume mediates improvements in hippocampal memory (Erickson et al., 2009, 2011; Chaddock et al., 2010). However, it is unknown whether these associations extend to breast cancer survivors. In rodents, voluntary wheel running following chemotherapy or radiation has been found to result in restoration of cognitive function (Fardell et al., 2012), hippocampal neurogenesis (Naylor et al., 2008), levels of precursor cells (Naylor et al., 2008), and neurotrophic factors such as BDNF, insulin-like growth factor 1 (IGF-1), and vascular endothelial growth factor (VEGF; Wong-Goodrich et al., 2010). Thus, CRF may hold promise for improving brain and cognitive health of human cancer survivors following primary treatment.

In the present study, we predicted that CRF would buffer declines in hippocampal volume in women within 3 years of completion of primary cancer treatment (surgery, chemotherapy and/or radiation), such that breast cancer survivors with higher CRF would have comparable hippocampal volume to that of non-cancer control participants. We explored anterior and posterior subsections of the hippocampus to examine whether breast cancer status, and/or CRF, had selective effects on brain structure in breast cancer survivors. We also hypothesized that larger hippocampal volumes would be associated with better memory performance on a task that required the maintenance of a flexible and dynamic map of spatial information, a task considered to be dependent on the hippocampus (Watson et al., 2013). In particular, individuals with hippocampal amnesia have been found to display impaired performance on this task, such that they reversed, or “swapped,” the relative positions of learned item pairs during attempts to reconstruct object arrays (Watson et al., 2013). Here we explored whether structural differences in the hippocampus, both as a function of breast cancer status and CRF level, also predicted memory performance in terms of “swaps.”

## Materials and Methods

### Participants

Breast cancer survivors and non-cancer control participants were recruited from East-Central Illinois. To be eligible for the study, breast cancer survivors had to be within 3 years of completion of primary cancer treatment (surgery, chemotherapy and/or radiation; could still be on hormone therapy). Furthermore, participants with a history of stroke, transient ischemic attack, surgery that involved removal of brain tissue, a score ≤23 on the modified Mini-Mental Status Exam (MSSE), or current use of computer-based brain training games (e.g., Lumosity®, BrainHQ®) were excluded. The study was approved by the University of Illinois Institutional Review Board, and all participants provided informed consent. All participants also provided written consent from a physician prior to study enrollment indicating clearance to participate in both the CRF, cognitive, and MRI testing. The present study included 29 female breast cancer survivors (all non-Hispanics) and 27 female controls (25 non-Hispanics, 2 Hispanics).

### Cardiorespiratory Fitness Assessment

CRF was assessed via a submaximal graded exercise treadmill test (Naughton protocol; Wasserman et al., 1999). After each participant was fitted with a heart rate monitor, he/she walked on a treadmill while speed and/or grade increased in 2 min stages until achieving 85% of a pre-determined, age-predicted maximum (220 age) heart rate. During each 2 min stage, heart rate, blood pressure, and subjective rating of perceived exertion were recorded. A 3 min monitored cool-down period of slower walking and a final 2 min monitored seated resting period were also included. CRF was derived from the treadmill test as an estimated V0_2 peak_ (American College of Sports Medicine, 2013).

### Structural MRI Protocol

High resolution T1-weighted brain images were acquired using a 3D Magnetization Prepared Rapid Gradient Echo Imaging (MPRAGE) protocol with 192 contiguous axial slices, collected in ascending fashion parallel to the anterior and posterior commissures, echo time (TE) = 2.32 ms, repetition time (TR) = 1900 ms, field of view (FOV) = 230 mm, acquisition matrix 256 mm × 256 mm, slice thickness = 0.90 mm, and flip angle = 9°. All images were collected on a Siemens Magnetom Trio 3T whole-body MRI scanner.

### FMRIB’s Integrated Registration and Segmentation Tool

Segmentation and volumetric analysis of the hippocampus were performed using a semi-automated, model-based subcortical tool (FMRIB’s Integrated Registration and Segmentation Tool; FIRST) in FMRIB’s Software Library (FSL) version 4.1.9 (Patenaude, 2007).

A two-stage affine registration to a standard space template (MNI space) with 1 mm resolution using 12-degrees of freedom and a subcortical mask to exclude voxels outside the subcortical regions was first performed on each participant’s MPRAGE. Next, the hippocampus was segmented with 30 modes of variation. To achieve accurate segmentation, the FIRST methodology models 317 manually segmented and labeled T1-brain images from normal children, adults, and pathological populations (obtained from the Center for Morphometric Analysis, Massachusetts General Hospital, Boston) as a point distribution model with the geometry and variation of the shape of each structure submitted as priors. Volumetric labels are parameterized by a 3D deformation of a surface model based on multivariate Gaussian assumptions. FIRST searches through linear combinations of shape modes of variation for the most probable shape (i.e., brain structure) given the intensity distribution in the T1-weighted image, and specific brain regions are extracted (see Patenaude et al., 2007a, b for further description of the method). Modes of variation are optimized based on leave-one-out cross-validation on the training set, and they increase the robustness and reliability of the results (Patenaude et al., 2007b). The segmentations were visually checked for errors. Finally, boundary correction was run, a process that classifies boundary voxels as belonging to the structure (or not) based on a statistical probability (*z*-score >3.00; *p* < 0.001). The volume of each participant’s brain region was measured in mm^3^ and converted to cm^3^ for presentation.

Anterior and posterior sections of the hippocampus were calculated by determining the center of gravity for both the left and right hippocampus for each participant. The *y* coordinate from the center-of-gravity calculation was used to divide the region into anterior and posterior sections, and the left and right volume of each anterior and posterior subsection was determined (Erickson et al., 2011).

### Spatial Memory Task

**Figure 1:**
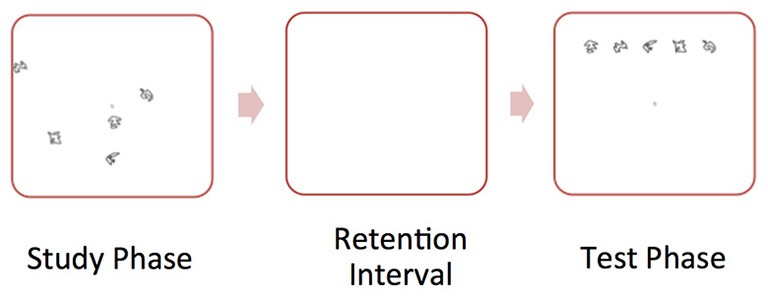
**Illustration of sample “study” and “test” trial of the spatial memory task**. During the “study” phase, the participant studied an object array. Then, the objects disappeared and appeared aligned at the top of the screen. During the “test” phase, the participant was told to use the computer mouse to reconstruct the original spatial layout of the objects.

We administered a spatial reconstruction task (Huttenlocher and Presson, 1979; Smith and Milner, 1981; Jeneson et al., 2010) to all participants. During each trial the participant studied an object array (containing five objects) and clicked on each object. Then, the objects disappeared and appeared aligned at the top of the screen. The participant was told to use the computer mouse to reconstruct the original spatial layout of the objects. Participants completed 25 trials. Figure 1 illustrates a “study” and “test” trial of the spatial memory task.

We focused on errors made in reconstructing the relative positions of objects, known as “swaps.” “Swaps” occur when objects switch positions between study and reconstruction (i.e., when the correct locations were filled but with misassignment of particular objects to particular locations). Hippocampal amnesics have been found to show more “swaps” on this task, thereby failing at binding item identities to locations (Watson et al., 2013). We report “swaps” as the rate that a swap occurs (per pair of items).

### Data Analysis

Univariate ANCOVAs were conducted to compare hippocampal volume and memory performance as a function of group (breast cancer survivor, non-cancer control), with age as a covariate. Given the small sample size and exploratory nature of this study, we explored associations between CRF and hippocampal volume stratified by group. We also performed a median split based on fitness level in the cancer participants and control participants to compare hippocampal volumes between the higher fit and lower fit cancer survivors and the non-cancer controls using independent *t*-tests. That is, one median split stratified the breast cancer survivors into two groups: (1) a lower fit breast cancer group with CRF levels lower than, and including, the median; and (2) a higher fit breast cancer group, with CRF levels above the median. A median split analysis was also conducted for the non-cancer control group to create a lower fit and higher fit control group.

Effect sizes (Cohen’s *d*) were calculated and reported (Cohen, 2013). Cohen’s *d* is a measure of the distance between two means, measured in standard deviations (SD). The following was the formula used to calculate the Cohen’s *d*: *d* = (Mean_1_−Mean_2_)/SD_pooled_, SD_pooled_ is the pooled standard deviation for the samples ${\text{SD}}_{\text{pooled}}\text{=}\sqrt{\left[({\text{SD}}_{\text{1}}^{\text{2}}{\text{+SD}}_{\text{2}}^{\text{2}})\text{/2}\right]}$.

## Results

### Participant Characteristics

The present study included 29 female survivors of primary (stage I-IIIA) breast cancer who underwent surgery, adjuvant chemotherapy treatment and/or radiation treatment (*N* = 11 radiation only, *N* = 7 chemotherapy only, *N* = 11 radiation and chemotherapy). On average, the participants were 17 ± 9 months off-therapy (range = 2–33 months). Survivors were all disease and relapse free at the time of evaluation. We also included 27 non-cancer female controls. There were no significant differences between the groups in terms of age (range = 41–73 years), years of education, CRF or MSSE (all *t* < 0.5, *p* > 0.5; Table 1).

**Table 1:** **Participant mean demographic, fitness, hippocampal volume, and memory data (SE) by group (breast cancer survivors, non-cancer controls; including *p*-values of independent *t*-tests and Cohen’s *d*)**.

Variable	Cancer	Control	Significance (*p*)	Cohen’s *d*
Age (years)	55.55 (1.48)	55.44 (2.13)	0.97	−0.01
Education (years)	16.56 (0.51)	16.64 (0.59)	0.28	0.28
VO_2_ max (mL/kg/min)	22.42 (1.00)	23.52 (1.01)	0.62	0.13
Mini-Mental Status Exam (MSSE)	29.28 (0.17)	29.04 (0.26)	0.44	0.21
Body Mass Index (BMI; kg/m^2^)	27.20 (0.88)	28.30 (1.18)	0.45	−0.20
Total Hippocampal Volume (cm^3^)	7.30 (0.13)	7.59 (0.11)	0.11	1.35
Anterior Hippocampal Volume (cm^3^)	4.22 (0.07)	4.36 (0.07)	0.18	0.36
Posterior Hippocampal Volume (cm^3^)	3.07 (0.06)	3.22 (0.05)	0.06	0.36
Left Anterior Hippocampal Volume (cm^3^)	2.09 (0.04)	2.16 (0.03)	0.18	0.36
Right Anterior Hippocampal Volume (cm^3^)	2.13 (0.04)	2.20 (0.04)	0.24	0.27
Left Posterior Hippocampal Volume (cm^3^)	1.53 (0.03)*	1.62 (0.02)*	0.02	0.62
Right Posterior Hippocampal Volume (cm^3^)	1.53 (0.03)	1.59 (0.03)	0.19	0.35
Memory “swap” errors (expected probability)	0.085 (0.01)	0.067 (0.01)	0.29	0.24

*Significantly different at p < 0.05.

### Group, Hippocampal Volume, and Memory

All hippocampal volumes for breast cancer survivors and non-cancer control participants are shown in Table 1. Radiation only, chemotherapy only, and radiation and chemotherapy groups were combined into a “breast cancer survivor” group given no significant differences in total hippocampal volume (*t* < 1.8, *p* > 0.05) between specific cancer treatment groups. Breast cancer survivors had significantly smaller volumes in the left posterior hippocampus compared to non-cancer control participants (Table 1; *F*_(1,53)_ = 5.48, *p* = 0.02; when controlling for age, which was associated with hippocampal volume, *r* = −0.273, *p* = 0.04). No group differences were found for anterior hippocampal volumes (all *F* < 1.9, *p* > 0.1) or total hippocampal volume (*F*_(1,53)_ = 2.78, *p* = 0.10).

**Figure 2:**
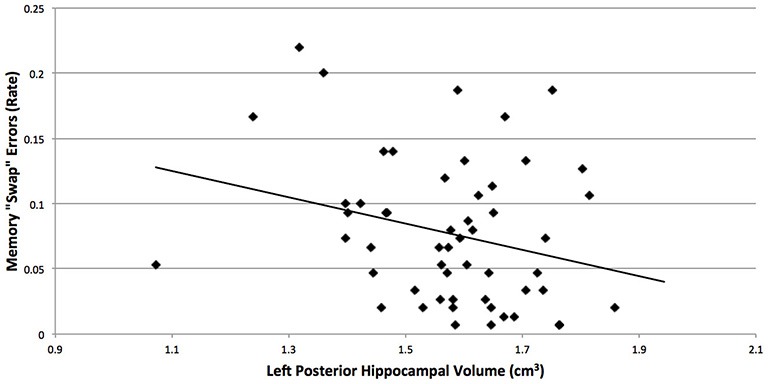
**More “swap” memory errors were associated with smaller left posterior hippocampal volumes (*r* = −0.27, *p* = 0.048), where the breast cancer survivors and non-cancer control participants differed in brain volume**. “Swaps” occur when objects switch positions between study and reconstruction, and hippocampal amnesics have been found to show more “swaps” on this task (Watson et al., 2013).

Although we predicted that breast cancer survivors would show more “swaps” on the spatial memory task, memory performance did not significantly differ between the groups (*t*_(56)_ = 1.2, *p* = 0.2). An examination of the means suggest more “swaps” in the cancer patients (*M* = 0.08, SD = 0.06) compared to control participants (*M* = 0.06, SD = 0.05; Cohen’s *d* = 0.36). Additionally, more “swap” memory errors were associated with smaller left posterior hippocampal volumes (*r* = −0.27, *p* = 0.048; Figure 2), where the groups differed in brain volume.

### Cardiorespiratory Fitness

**Figure 3:**
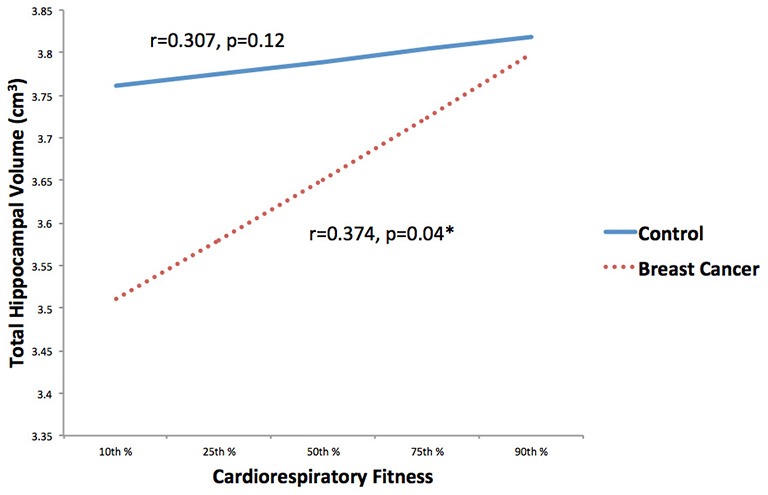
**Associations between CRF and hippocampal volume stratified by group (breast cancer survivor, non-cancer control) were explored**. CRF and total hippocampal volume were positively correlated in the cancer survivors, but not the control group. *p < 0.05.

CRF and total hippocampal volume were positively correlated (*r* = 0.351, *p* = 0.008). Within condition analyses indicated that this relationship remained significant in the cancer survivors (*r* = 0.374, *p* = 0.04), but not the control group (*r* = 0.307, *p* = 0.119; Figure 3).

**Figure 4:**
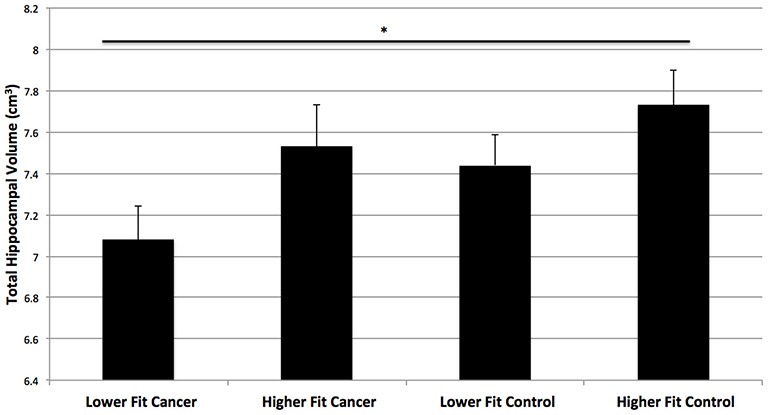
**Hippocampal volumes of higher fit and lower fit cancer survivors and higher fit and lower fit control participants (as determined by a median split)**. Higher fit cancer survivors did not show significant differences in hippocampal volume compared to lower fit or higher fit non-cancer control participants (*p* > 0.3). Lower fit cancer survivors showed significantly smaller total hippocampal volumes than higher fit control participants (*p = 0.01).

Next, we compared the hippocampal volumes of dichotomized higher fit and lower fit cancer survivors to the higher fit and lower fit control participants (Table 2; Figure 4). As predicted, higher fit cancer survivors did not show significant differences in total, anterior, and posterior hippocampal volume compared to lower fit or higher fit non-cancer control participants (all *t* < 0.9, all *p* > 0.3). Lower fit cancer survivors showed significantly smaller hippocampal volumes than higher fit control participants—in terms of total hippocampal volume (*t*_(26)_ = 2.80, *p* = 0.010), anterior hippocampal volume (*t*_(26)_ = 2.65, *p* = 0.014), and posterior hippocampal volume (*t*_(26)_ = 2.79, *p* = 0.010). Lower fit cancer survivors also showed significantly smaller left posterior hippocampal volumes compared to lower fit control participants (*t*_(27)_ = 2.39, *p* = 0.024). An effect size (Cohen’s *d*) of 0.87 suggested a large effect for the difference in total hippocampal volume between lower fit cancer survivors and control participants, with a negligible effect size (Cohen’s *d* = 0.13) for the difference in total hippocampal volume between higher fit cancer survivors and control participants.

**Table 2:** **Participant mean demographic, fitness, hippocampal volume, and memory data (SE) by median split group (lower fit and higher fit breast cancer survivors, lower fit and higher fit non-cancer controls)**.

Variable	Lower fit cancer	Higher fit cancer	Lower fit control	Higher fit control
Age (years)	56.93 (1.54)	54.07 (2.59)	56.93 (1.54)	49.77 (3.07)
VO_2_ max (mL/kg/min)	18.10 (0.77)	27.05 (0.80)	19.48 (0.78)	27.03 (1.12)
Total Hippocampal Volume (cm^3^)	7.08 (0.16)*	7.53 (0.20)	7.44 (0.15)	7.73 (0.17)*
Anterior Hippocampal Volume (cm^3^)	4.10 (0.09)*	4.37 (0.11)	4.28 (0.09)	4.46 (0.10)*
Posterior Hippocampal Volume (cm^3^)	2.98 (0.07)*	3.16 (0.09)	3.17 (0.07)	3.28 (0.07)*
Left Anterior Hippocampal Volume (cm^3^)	2.02 (0.05)*	2.18 (0.05)	2.12 (0.04)	2.21 (0.05)*
Right Anterior Hippocampal Volume (cm^3^)	2.08 (0.05)*	2.19 (0.06)	2.16 (0.06)	2.25 (0.07)*
Left Posterior Hippocampal Volume (cm^3^)	1.49 (0.04)^*⧫^	1.59 (0.04)	1.61 (0.03)*	1.65 (0.04)^⧫^
Right Posterior Hippocampal Volume (cm^3^)	1.50 (0.04)*	1.57 (0.06)	1.56 (0.04)	1.63 (0.04)*
Memory “swap” errors (expected probability)	0.092 (0.017)	0.076 (0.013)	0.076 (0.014)	0.059 (0.015)

*Groups are significantly different at *p* < 0.05 for specific variable (row) of interest; ^⧫^Groups are significantly different at *p* < 0.05 for specific variable (row) of interest.

CRF was not correlated with “swaps” on the spatial memory task in the cancer survivors (*r* = −0.024, *p* = 0.9) or control participants (*r* = −0.351, *p* = 0.07). Additionally, higher fit and lower fit cancer survivors did not differ from higher fit and lower fit control participants in terms of “swaps” (Table 2).

## Discussion

As one in eight women will develop breast cancer over the course of her lifetime (DeSantis et al., 2014), understanding changes in brain and cognitive health following cancer treatment, as well as approaches to improve cancer recovery, are of public health importance. The present study is the first to report that higher fit breast cancer survivors did not show significant differences in hippocampal volume compared to non-cancer control participants, whereas lower fit breast cancer survivors showed significantly smaller hippocampal volumes compared to both lower fit and higher fit control participants. Consequently, these results raise the possibility that the protective effects of CRF on hippocampal structure in healthy adults (Erickson et al., 2009; Bugg and Head, 2011) extend to cancer survivors.

The present study also supports previous findings of smaller hippocampal volumes in breast cancer survivors particularly in the left (Nakano et al., 2002; Kesler et al., 2013a) and posterior (Bergouignan et al., 2011) hippocampus. Further, despite no significant group differences on the memory task (Cohen’s *d* = 0.36), smaller left posterior hippocampal volumes were associated with poorer memory performance on a spatial learning task of hippocampal function (as also suggested in Bergouignan et al., 2011). It is interesting to note that although only left posterior hippocampal volume differed between cancer and control groups, CRF helped offset hippocampal volume declines across the total hippocampus, in both anterior and posterior subsections. Aerobic exercise has been found to selectively increase the volume of the anterior hippocampus that includes the dentate gyrus, where cell proliferation occurs (van Praag et al., 2005; Li et al., 2008; Creer et al., 2010; Erickson et al., 2011). The posterior hippocampus has not yet been linked to CRF or exercise, but this area is known to play a role in spatial learning and memory (Moser et al., 1993; Colombo et al., 1998; Maguire et al., 2000; Bannerman et al., 2004), which are cognitive abilities influenced by CRF (Erickson et al., 2009) and aerobic exercise (Cotman and Berchtold, 2002; Erickson et al., 2011). The posterior hippocampus also plays a role in depression and post-traumatic stress disorders, two conditions found in cancer survivors (de Geus et al., 2007; Maller et al., 2007; Bonne et al., 2008; Bergouignan et al., 2011).

Although promising, these results must be viewed cautiously given our *a priori* predictions and the relatively small sample size. Nevertheless, the effect sizes (Cohen’s *d* = 0.87 [difference in total hippocampal volume between lower fit cancer survivors and control participants], and Cohen’s *d* = 0.13 [difference in total hippocampal volume between higher fit cancer survivors and control participants]) suggest the importance of investigating how individual differences in CRF play a role in brain changes of breast cancer survivors. The results also parallel animal research demonstrating that wheel running following chemotherapy or radiation (one week to one month post-treatment) restores hippocampal brain health (Naylor et al., 2008; Wong-Goodrich et al., 2010; Fardell et al., 2012). As cancer treatments such as chemotherapy and radiation are known to target cell proliferation, neurogenesis, and vascularization in the hippocampus (Pereira Dias et al., 2014), the same cascades known to relate to CRF in rodent models of wheel running, it seems possible that CRF may play a role in these brain changes following cancer treatment in humans too.

The present investigation provides a foundation for exploring associations among CRF, hippocampal volume, and memory in cancer survivors and supports the need for randomized, controlled trials to establish causal associations among participation in physical activity aimed to improve CRF, brain structure and cognition in cancer patients. It would be interesting to collect CRF and hippocampal volume data before, during, and after breast cancer therapy to understand how indices of physical and cognitive health change from cancer diagnosis to post-treatment. Unfortunately, older breast cancer patients (age = 55 years) have been found to show impaired CRF (e.g., 27% lower than age-matched controls) across the survivorship continuum (Jones et al., 2012; Lakoski et al., 2013), further emphasizing the importance of investigating the role of physical activity during cancer recovery. In fact, as a variety of lifestyle and health-related behaviors influence hippocampal size and function (e.g., sleep, nutrition), it would be interesting to explore the role of other lifestyle factors in cancer recovery too (Pereira Dias et al., 2014).

Future work may also investigate how CRF moderates other structural and functional brain changes following cancer treatment. In addition to changes in hippocampal volume following cancer treatment, post-treatment declines in gray matter volume (de Ruiter et al., 2012), white matter tract integrity (Abraham et al., 2008; Deprez et al., 2011), white matter hyperintensities (Ferguson et al., 2007) as well as altered brain activation during memory tasks (Kesler et al., 2009; de Ruiter et al., 2011) and resting state networks (Bruno et al., 2012; Kesler et al., 2013b) have been found to account for cancer-related cognitive declines. Whether higher levels of CRF are protective against these brain changes remains to be determined. Moreover, as our sample was heterogeneous in terms of disease, host and treatment variables, which is typical across the literature, it would be interesting to explore how specific treatment regimens are associated with fitness-related changes in brain structure.

In conclusion, the present study is the first to identify that CRF holds promise for improving the brain health of breast cancer survivors within 3 years of completion of treatment. Our findings also support further research on the inclusion of physical activity, aimed to improve CRF, in treatment protocols for breast cancer survivors.

## Conflict of Interest Statement

The authors declare that the research was conducted in the absence of any commercial or financial relationships that could be construed as a potential conflict of interest.
